# Can knowledge-based DVH predictions be used for automated, individualized quality assurance of radiotherapy treatment plans?

**DOI:** 10.1186/s13014-015-0542-1

**Published:** 2015-11-19

**Authors:** Jim P. Tol, Max Dahele, Alexander R. Delaney, Ben J. Slotman, Wilko F. A. R. Verbakel

**Affiliations:** Department of Radiotherapy, VU University Medical Center, De Boelelaan 1117, 1081 HV Amsterdam, The Netherlands

**Keywords:** Treatment planning quality assurance, OAR sparing, Knowledge-based planning

## Abstract

**Background:**

Treatment plan quality assurance (QA) is important for clinical studies and for institutions aiming to generate near-optimal individualized treatment plans. However, determining how good a given plan is for that particular patient (individualized patient/plan QA, in contrast to running through a checklist of generic QA parameters applied to all patients) is difficult, time consuming and operator-dependent. We therefore evaluated the potential of RapidPlan, a commercial knowledge-based planning solution, to automate this process, by predicting achievable OAR doses for individual patients based on a model library consisting of historical plans with a range of organ-at-risk (OAR) to planning target volume (PTV) geometries and dosimetries.

**Methods:**

A 90-plan RapidPlan model, generated using previously created automatic interactively optimized (AIO) plans, was used to predict achievable OAR dose-volume histograms (DVHs) for the parotid glands, submandibular glands, individual swallowing muscles and oral cavities of 20 head and neck cancer (HNC) patients using a volumetric modulated (RapidArc) simultaneous integrated boost technique. Predicted mean OAR doses were compared with mean doses achieved when RapidPlan was used to make a new plan. Differences between the achieved and predicted DVH-lines were analyzed. Finally, RapidPlan predictions were used to evaluate achieved OAR sparing of AIO and manual interactively optimized plans.

**Results:**

For all OARs, strong linear correlations (R^2^ = 0.94–0.99) were found between predicted and achieved mean doses. RapidPlan generally overestimated the amount of achievable sparing for OARs with a large degree of OAR-PTV overlap. RapidPlan QA using predicted doses alone identified that for 50 % (10/20) of the manually optimized plans, sparing of the composite salivary glands, oral cavity or composite swallowing muscles could be improved by at least 3 Gy, 5 Gy or 7 Gy, respectively, while this was the case for 20 % (4/20) AIO plans. These predicted gains were validated by replanning the identified patients using RapidPlan.

**Conclusions:**

Strong correlations between predicted and achieved mean doses indicate that RapidPlan could accurately predict achievable mean doses. This shows the feasibility of using RapidPlan DVH prediction alone for automated individualized head and neck plan QA. This has applications in individual centers and clinical trials.

## Background

The increasing complexity of radiotherapy treatment planning, particularly due to the attempt to spare more individual organs-at-risk (OARs), has made it challenging to efficiently produce consistent, high quality radiotherapy treatment plans [1, 2]. This has led to considerable interest in (semi) automated planning strategies [3–9]. Plan quality assurance (QA) is another step in the treatment preparation workflow that might benefit from increased automation, since often, insufficient attention is given to evaluating whether a given plan can be improved. The time consuming [10], difficult and subjective nature of this process QA makes it hard to be confident that good plan quality is being obtained for individual patients. Furthermore, sub-optimal plans submitted to clinical trials have been correlated with worse clinical outcomes [11], suggesting that high quality plans are important for maximizing treatment outcomes. However, the fact that sub-optimal plans were accepted to the trial identifies a clear need for a robust and efficient plan QA tool to determine in near real-time whether plans meet an acceptable quality standard for individual patients [12]. Although Moore et al. [13] have recently published an analysis in which the number of patients at unnecessary risk for normal tissue complication probability was determined by comparing the predicted and achieved dosimetry, they used an in-house developed knowledge-based planning solution [14].

In contrast we have investigated whether OAR dose-volume histograms (DVH) predicted by RapidPlan™, a commercial knowledge-based planning solution (Varian Medical Systems, Palo Alto, USA), are accurate enough to serve as a plan QA tool that could be used in clinical departments or for clinical trials. RapidPlan utilizes a library of plans to construct a model [14–17]. This model uses the geometrical features and associated dosimetry of the plans included in the library to predict a range of achievable DVH-lines for OARs of new patients. This range consists of the mean estimated DVH-line ± one standard deviation. The accuracy of the DVH prediction is therefore influenced by the consistency of the plans and the range of patient geometries in the library. The DVH-lines obtained during treatment planning for a patient can be compared with the DVHs predicted by RapidPlan. If the RapidPlan predictions are accurate, this comparison can be used to determine whether the evaluated plan has achieved adequate OAR sparing, as judged against the model library. This approach does not require the creation of additional plans and it would therefore present a fast and straightforward solution for plan QA. Although there have been reports of improvements in OAR sparing using RapidPlan [5–7], these do not necessarily imply that there was a close correspondence between the achieved and predicted DVH-lines, nor has this relationship been evaluated.

Our work differs from previous publications [14, 18] in several ways, including (i) using a commercial program to predict achievable DVHs, (ii) use of complex head and neck cancer (HNC) treatment plans, involving sparing of the parotid glands, submandibular glands, oral cavity and individual swallowing muscles, along with two PTVs and a simultaneous integrated boost, (iii) detailed evaluation of the relationship between predicted and achieved dosimetry along the entire DVH-curve, and (iv) demonstration of QA application by using DVH predictions generated by RapidPlan to benchmark previously created plans.

## Methods

### General description of treatment planning

All HNC plans were created with a simultaneous integrated boost (SIB) technique using 6MV photons and 2 full RapidArc™ (Varian Medical Systems, Palo Alto, USA) arcs. In 35 fractions, plans aimed to deliver 95 % of the prescribed dose of 54.25Gy/70.0Gy to 98 %/99 % of the elective/boost PTV (PTV_E_/PTV_B_), while limiting the volume of each PTV receiving >107 % of the prescribed dose. A 5mm transition zone (PTV_T_) was created between PTV_E_ and PTV_B_ to facilitate dose fall-off between them. The optimization goals and included OARs have been outlined in detail previously [7, 19–21].

### Model library

**Fig. 1:**
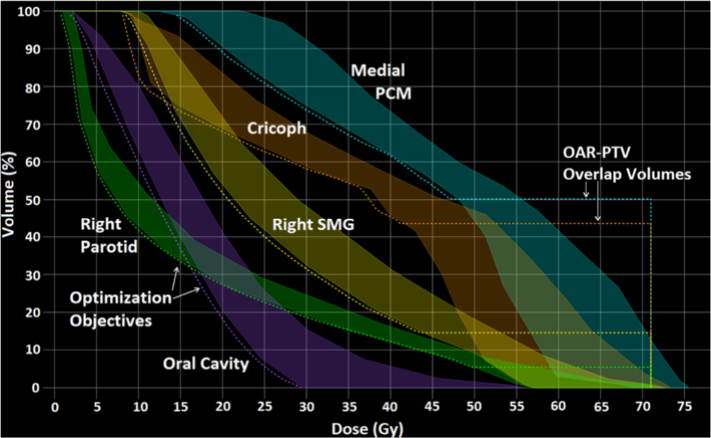
Organ-at-risk (OAR) dose-volume histogram (DVH) prediction ranges (shaded regions) generated by the RapidPlan model and optimization objectives placed along the inferior DVH prediction boundary (dotted lines). To prevent underdosing of the planning target volume (PTV), line objectives are placed horizontally in the portion of the OAR that overlaps with the PTV

Ninety HNC patients treated between 2012 and 2014 were arbitrarily selected. Primary tumor locations included the oropharynx (*n* = 47), (supra-)glottic larynx (*n* = 25), hypopharynx (*n* = 10), nasopharynx (*n* = 2), unknown (*n* = 2), thyroid (*n* = 1) and maxillary sinus (*n* = 1). Since the goal was to create a general HNC RapidPlan model, no differentiation was made regarding primary tumor location. New treatment plans for these patients were created using our in-house developed automatic interactive optimizer (AIO). AIO was developed to produce plans with more consistent OAR sparing than manually optimized plans by automatically adapting the dose-volume objectives throughout the interactive optimization process, ensuring that the same level of attention is given to sparing of each OAR [9]. These plans were created in the Eclipse treatment planning system using the progressive resolution optimizer (PRO) v10.0.28 and anisotropic analytical algorithm (AAA) with a 2.5mm grid. The geometric and dosimetric features of the AIO plans were used to create a RapidPlan model library. This model could then be used to predict achievable OAR DVHs for patients outside the model library, based on their OAR-PTV geometry (Table 1). RapidPlan automates the optimization process by generating a line of optimization objectives just below the inferior boundary of the OAR DVH prediction range. A standard set of optimization objective priorities, reflecting our institutional practice, was used for all patients. Figure 1 shows the RapidPlan optimization window with various OARs included. The shaded regions represent the OAR DVH prediction ranges, while the dotted lines represent the automatically generated optimization objectives, placed just below the inferior boundary of the DVH prediction range. To prevent underdosing of the PTVs, RapidPlan is designed to place the line objective horizontally in the OAR-PTV overlap volume. In this study, line objectives were generated for the parotid glands, submandibular glands, individual swallowing muscles and oral cavity, while fixed maximum point dose objectives of 37 Gy and 39 Gy were set for the spinal cord/brainstem and their planning at risk volumes, respectively.

**Table 1:** Summary of the RapidPlan model characteristics for each individual organ-at-risk (OAR)

Structure name	Number of structures included in model	Suggested number in model	Model fit (R^2^)	Regression model’s average χ^2^
Contralateral Parotid	90	-	0.77	1.096
Contralateral Submandibular	69	-	0.68	1.094
Ipsilateral Parotid	86	-	0.52	1.051
Cricopharyngeal Muscle	52	-	0.75	1.117
Lower Larynx	46	-	0.82	1.125
Upper Larynx	26	36	0.76	1.196
Inferior PCM^a^	38	48	0.86	1.229
Medial PCM^a^	24	42	0.58	1.169
Superior PCM^a^	39	48	0.89	1.128
Upper Esophageal Spincter	68	-	0.85	1.147
Oral Cavity	76	-	0.80	1.073

*Abbreviation*: ^a^ *PCM* Pharyngeal constrictor muscle

The model fit R^2^ value shows the correlation between the geometric and dosimetric regression parameters. Where relevant, the RapidPlan model configuration algorithm can suggest to include more OARs in the model library. This could for example be in the case where there is large variation in OAR sparing between the plans that are included in the model library. Including more plans could improve the prediction accuracy. Because not every structure was attempted to be spared in the original clinical plan, the number of structures included in the model varies per OAR

### Replanning of dosimetric outliers

The RapidPlan model configuration window provides detailed information regarding geometric and dosimetric outliers that are present in the created model. Although AIO provides an automated and consistent approach to OAR dose reduction during the optimization process, some OARs could still be identified by the model as providing insufficient sparing. This could for example be because a trade-off with sparing of other OARs or strict PTV dose homogeneity criteria prevented better sparing. The consistency of the plans included in the model was therefore improved by two iterations of replanning such dosimetric outliers. In this process, the included OAR DVHs were compared against the range of achievable DVHs predicted by the model. A patient was replanned using RapidPlan if, subjectively judged, a meaningful improvement in sparing was predicted for at least one OAR. The RapidPlan model configuration window assists the user in this process by allowing them to visualize the predicted and achieved DVHs for all OARs included in the model. Cleaning the model in this fashion is intended to improve the consistency of the included plans and lead to more accurate predictions of achievable OAR DVHs for new patients.

**Fig. 2:**
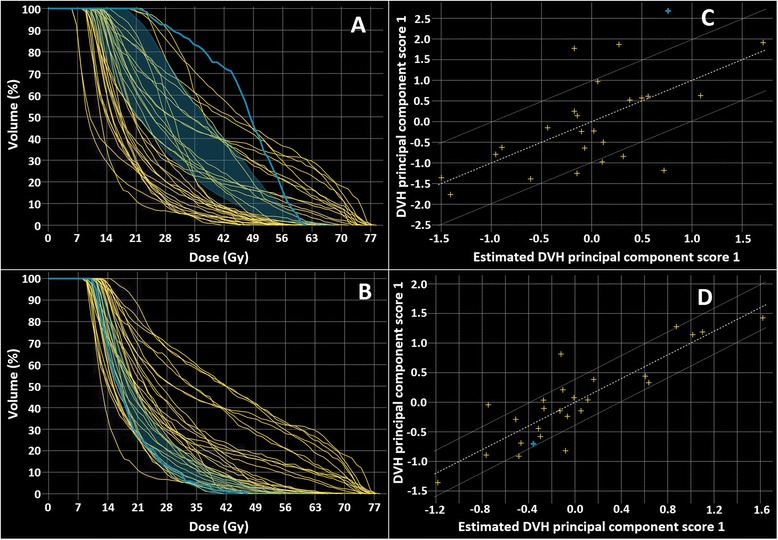
Example of an upper larynx contained in the RapidPlan model that was identified as a dosimetric outlier in the model configuration window. Screenshots taken from the RapidPlan model configuration window. The discrepancy between the predicted and achieved dose-volume histogram (DVH) lines (**a**) was solved by replanning the corresponding patient (indicated by solid blue line) using the RapidPlan model (**b**). The shaded region indicates the DVH prediction range for the selected plan. RapidPlan uses principal component analysis to decompose the shape of the achieved and predicted DVHs in the model library, allowing for a more consistent way to compare the estimated and obtained dosimetry. The residual plot (**c**) shows the correlation between the obtained (DVH principal component score 1) and predicted dosimetry (estimated DVH principal component score 1) after replanning (**d**), indicating that the predicted OAR DVH closely corresponds to the OAR DVH included in the model library. Since more than one upper larynx was identified as an outlier in the two iterations of outlier replanning, more DVHs are noted to change

**Fig. 3:**
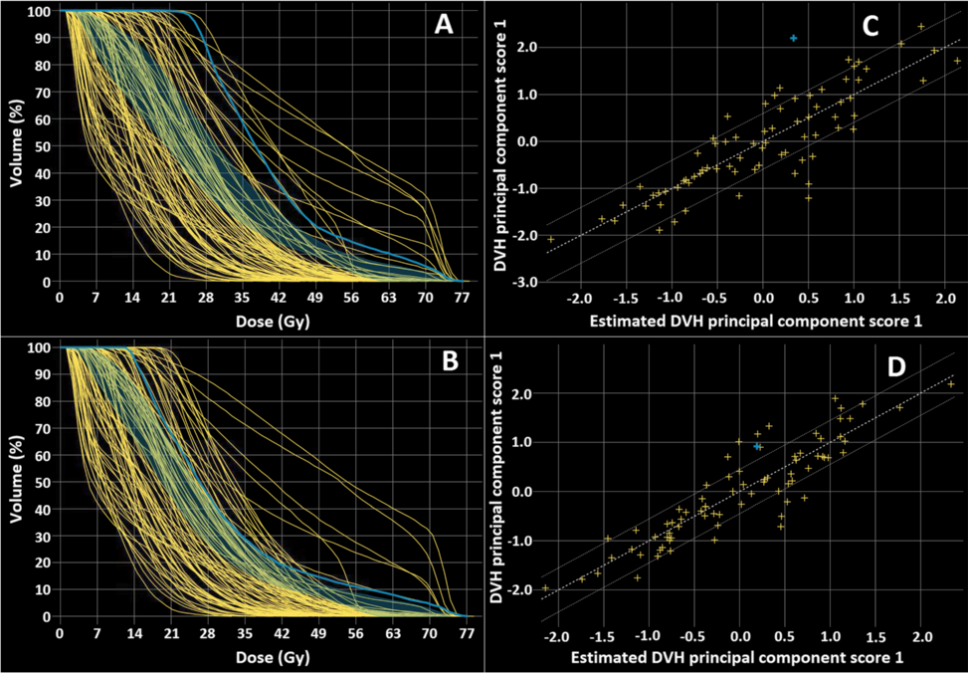
Similar to Fig. 2, an oral cavity identified as an outlier in the RapidPlan model library

As an example, Figs. 2a and 3a show two OARs identified as dosimetric outliers because of a discrepancy between the predicted DVH range (shaded blue region) and the achieved DVH (solid blue line), as visualized in the model configuration window. Figures 2b and 3b show the resulting DVH-lines after replanning the corresponding patients using the RapidPlan model, resulting in improved sparing for both OARs and a closer correspondence between the predicted and achieved DVHs. The residual plots (Figs. 2c and 3c), indicating the relation between the achieved OAR DVH (y-axis) and the OAR DVH predicted by the RapidPlan model (x-axis), also improved after replanning (2d and 3d). In total, 19 and 15 plans containing one or more dosimetric outlier OARs were replanned in the first and second iteration, respectively.

### Evaluation group for plan quality checking

The evaluation dataset consisted of the contoured planning CT-scans of 20 HNC patients treated between 2012 and 2013, along with their manual interactively optimized clinical plans (i.e. plans where the planner manually adapted the position of the OAR dose-volume objectives during the optimization process, relative to the position of the DVH-lines). For the evaluation group, primary tumors were oropharynx (*n* = 11), (supra-)glottic larynx (*n* = 5), hypopharynx (*n* = 2), and unknown (*n* = 2). These plans were not part of the model library. The following investigations were performed:

1. For each OAR spared in the original clinical plan, the mean dose predicted by the RapidPlan model was derived by creating a “mid-prediction” DVH-line running through the middle of the DVH prediction range (Fig. 4). To determine the accuracy of the predicted DVH, this mean dose was compared against the mean dose that was obtained when a plan was made using the RapidPlan model.

**Fig. 4:**
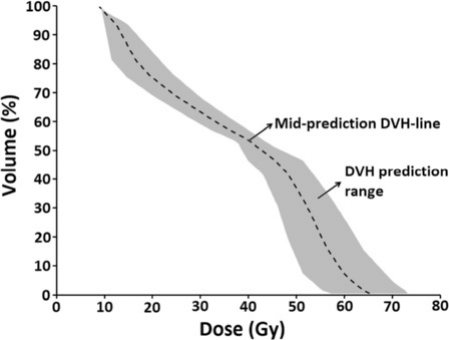
The mid-prediction dose-volume histogram (DVH) line (dashed) running through the middle of the DVH prediction range (shaded region). This was used as a surrogate for the prediction DVH in this study and determined using an in-house developed program coded in Lazarus (http://www.lazarus.freepascal.org/)
2. Because this comparison of mean doses does not reflect possible differences between the mid-prediction DVH-line and the achieved DVH-line, this difference was determined over the entire dose range.
3. The mid-prediction DVH-line was used to evaluate the quality of AIO plans and manually optimized clinical plans for the 20 patients in the evaluation group. The manually optimized plans were created before AIO was introduced in our clinic, and based on prior experience [7], were generally expected to provide less OAR sparing than the AIO plans. For illustrative purposes, the plans were considered acceptable if the mean dose to the composite (volume weighted) salivary glands, oral cavity and composite swallowing muscles was no more than 3 Gy, 5 Gy and 7 Gy higher, respectively, than the mean dose of the mid-prediction DVH-line. These arbitrary thresholds were chosen to represent clinically meaningful values. A higher threshold value was used for the oral cavity because institutional experience suggest that this structure is subject to more contouring and geometric variability. The threshold was highest for the swallowing muscles because these structures are relatively small and large dose differences are more easily obtained when replanning. Since the OAR DVH prediction by the RapidPlan model takes the geometrical features of the evaluated patient into account, this provides a patient specific approach to plan quality assurance.

## Results

**Fig. 5:**
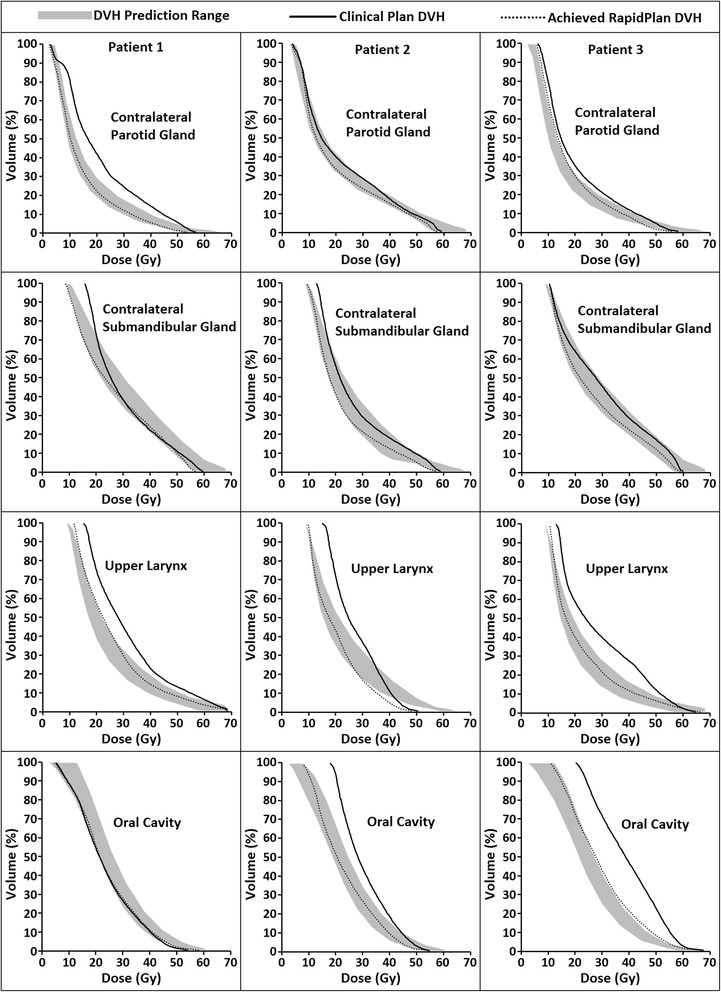
Examples of predicted dose-volume histogram (DVH) ranges (shaded regions) and achieved DVH-lines for multiple organs-at-risk (OARs) of three patients. The solid lines represent the DVHs that were achieved in the previously created clinical plans, while the dotted lines indicate the DVHs that were obtained when the RapidPlan model was used to create a new treatment plan. The mid-prediction DVH-line used for the analysis in the present report is located in the middle of the shaded region

Figure 5 shows for the first 3 patients examples of the DVH prediction range estimated by the RapidPlan model along with the DVHs that were achieved when using RapidPlan to create a new treatment plan and the previously created clinical plan DVHs. For the contralateral parotid and submandibular glands, the achieved DVHs were relatively comparable for both planning methods, indicating that the clinical plan spared these structures comparably to the plans that were included in the model. More variation is seen for the upper larynx and oral cavity, where the predicted gains generally were achieved by RapidPlan. The time required to generate the DVH predictions was less than 2 min while the optimization time of the RapidPlan plans was typically 10-15 min.

**Fig. 6:**
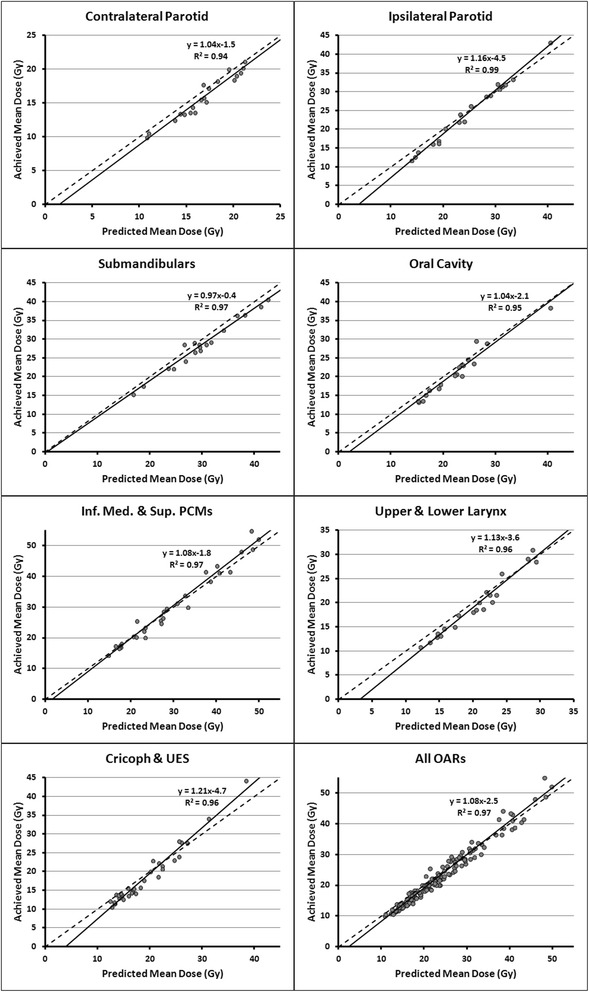
For multiple organs-at-risk (OARs), the correlation between predicted (x-axis) and achieved (y-axis) mean OAR doses. The solid lines represent fits created through all datapoints, while the dashed line indicates a linear fit through the origin. The R^2^ values indicates the goodness-of-fit of the solid line with the datapoints. For conciseness, some individual swallowing muscles and the contralateral, and ipsilateral submandibular gland are analyzed together in these graphs. The number of OARs included in these graphs can vary depending on whether they were designated to be spared in the original clinical plan

The maximum dose values to the spinal cord and brainstem were found to be clinically acceptable in all plans. Compared to the manually optimized plans, RapidPlan improved mean composite salivary gland doses by on average 2.0 ± 2.1Gy (range of -2.7 Gy to 6.5 Gy), mean oral cavity doses by 3.6 ± 3.6Gy (-2.1 Gy to 9.7 Gy) and mean composite swallowing muscle doses by 5.9 ± 2.9 Gy (0.6 Gy to 11.4 Gy). Figure 6 shows the predicted mean dose plotted against the achieved mean dose for multiple OARs. The solid line represents a linear fit created through all datapoints. The dashed line has a slope of 1 and runs through the origin, meaning that for OARs on this line, the mean dose that was predicted by RapidPlan was exactly achieved. All OARs showed a strong linear correlation between predicted and achieved mean doses, with R^2^ correlation coefficient values ranging from 0.94 for the contralateral parotid gland to 0.99 for the ipsilateral parotid gland. For all OARs combined, the linear fit has a R^2^ of 0.97, and a slope greater than 1 (1.08). This indicates that on average, the achieved mean dose was slightly higher than the mean dose that was predicted by RapidPlan. This was likely caused by several OARs with mean doses >40 Gy that were located above the dashed fits, indicating that the RapidPlan model overestimated the amount of OAR sparing that could be achieved. These OARs consisted of one ipsilateral parotid gland, one cricopharyngeal muscle and 6 pharyngeal constrictor muscles, overlapping with the PTVs by 43 ± 11 %, on average. For these OARs, RapidPlan was unable to accurately predict the amount of OAR sparing that was achieved in the portion of the OAR that overlapped with the elective and boost PTV.

**Fig. 7:**
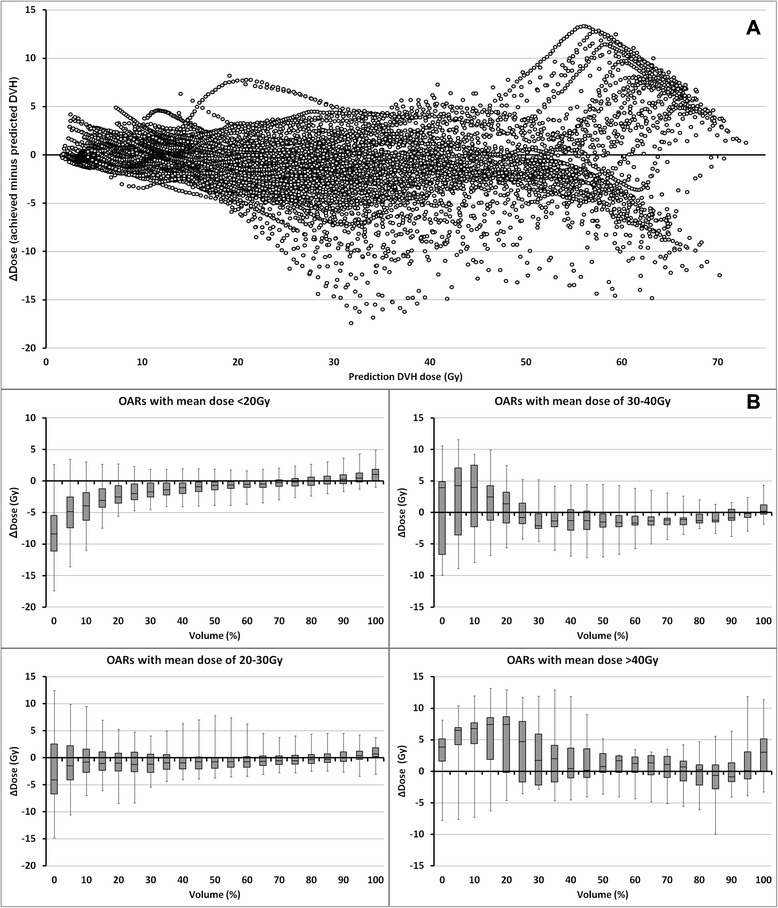
The prediction accuracy of RapidPlan along the dose-volume histogram (DVH) line. **a** Dose difference between the achieved and predicted DVH-line (ΔDose, y-axis) plotted against the dose of the predicted DVH-line (x-axis). **b** Box-whisker plots of ΔDose as a function of organ-at-risk (OAR) volume (x-axis) for four different ranges of OAR mean doses. Lower OAR volumes are typically associated with high doses

For each OAR, Fig. 7a shows the dose difference (ΔDose, y-axis), computed as achieved DVH dose minus mid-prediction DVH dose, plotted against the mid-prediction DVH dose (x-axis). Series of datapoints can be noted running through the graph. These datapoints belong to OARs for which ΔDose changes gradually with dose. More sudden, larger changes in ΔDose, suggesting less accurate predictions, can also be noted, for example in the bottom portion of the graph around 30-40 Gy. This graph shows that the prediction is generally more accurate at low OAR doses. At high OAR doses, and therefore at large overlap volumes with the PTVs, RapidPlan often overestimated the amount of sparing that could be achieved (ΔDose values greater than 0). Figure 7b shows the same ΔDose values at 5 % OAR volume increments, separated on the basis of the mean OAR dose achieved in the final RapidPlan plan. For all OARs, ΔDose is closer to 0 at higher OAR volumes (typically receiving low doses). At the higher dose regions (OAR volumes <30 %), the amount of achievable sparing is underestimated for OARs with mean doses <20 Gy while it is progressively overestimated for OARs with higher mean doses. This likely resulted from the horizontal placement of the dose-volume objectives at the OAR-PTV overlap volume (Fig. 1). Consistent with this hypothesis, the amount of OAR-PTV overlap for the OARs with achieved mean doses of <20 Gy, 20-30 Gy, 30-40 Gy and >40 Gy was 1.2 ± 2.0 %, 7.7 ± 7.0 %, 19.8 ± 9.8 % and 35.9 ± 11.9 %, respectively.

**Fig. 8:**
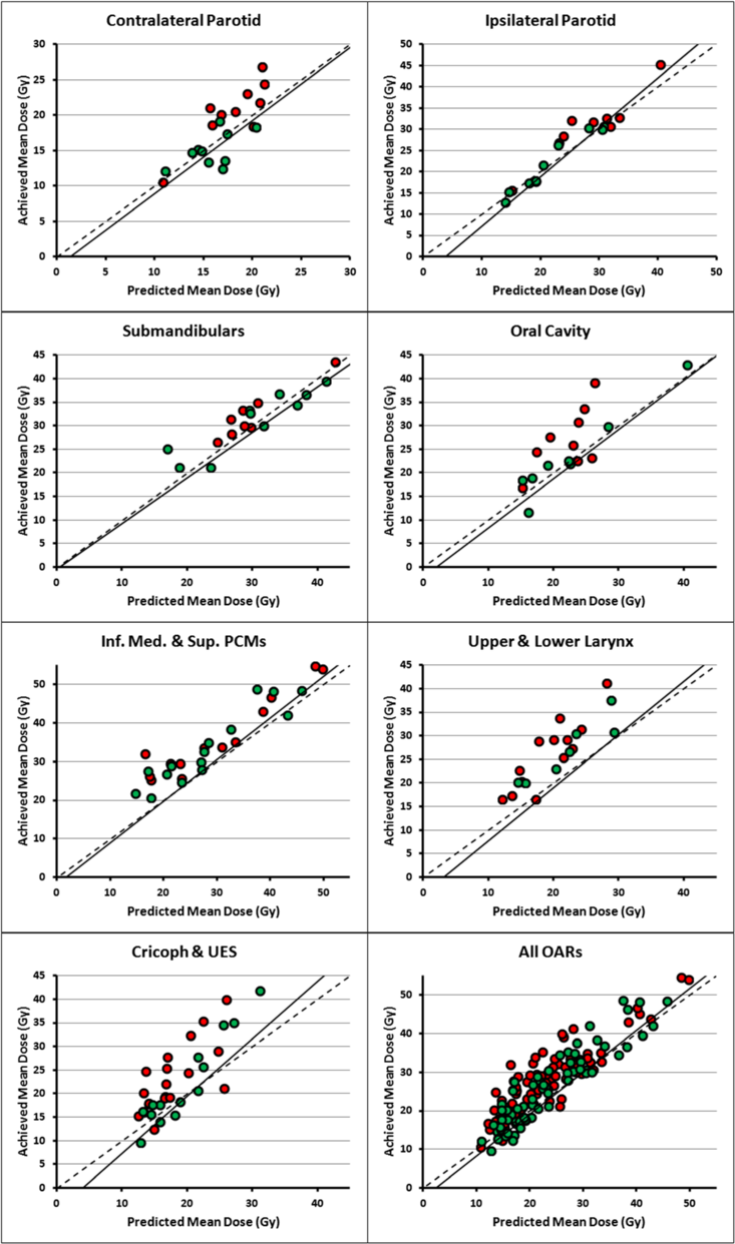
The organs-at-risk (OARs) of the clinical plans that passed (green circles) and failed (red circles) the evaluation criteria, along with the linear fit between the RapidPlan predicted and achieved mean dose found in Fig. 6. Thresholds of 3 Gy, 5 Gy and 7 Gy were used for quality evaluation of the composite (volume-weighted) salivary glands, oral cavity and composite swallowing muscles, respectively. Datapoints of individual OARs with similar predicted and achieved mean doses could still fail the criteria because the analysis was done based on composite OARs

The OAR DVH predictions generated by the RapidPlan model were used to re-evaluate the quality of the 20 manually optimized clinical plans, benchmarked using the mean dose of the mid-prediction DVH-line. 10 plans were found acceptable and provided sparing of the composite salivary glands, oral cavity and composite swallowing muscles at most 3 Gy, 5 Gy and 7 Gy higher than the mean dose of the mid-prediction DVH-line generated for these structures, respectively. Figure 8 shows the predicted and achieved mean dose values of the clinical plans that passed (green circles) and failed (red circles) these criteria, combined with the linear fits between predicted and achieved RapidPlan mean dose shown in Fig. 6. The small number of points located below the fit indicate that for the majority of OARs, the RapidPlan model improved OAR sparing over the clinical plan. Using the same thresholds, 4 AIO plans did not pass the QA, all because of inferior oral cavity sparing. The salivary glands and swallowing muscles were within the chosen thresholds for OAR sparing in all AIO plans. As expected from the high R^2^ values, after replanning of the patients that violated the criteria using RapidPlan, all resulting OAR mean doses fell within the respective threshold range from the predicted mean doses.

## Discussion

This study assessed the potential to use OAR DVH predictions generated by a RapidPlan model to evaluate the dosimetric quality of plans that were not included in the model. For all OARs, strong correlations were found between mean OAR doses predicted by the model and mean doses achieved after the model was used to guide the creation of a new treatment plan, with linear fit slopes close to one. This means that in general, achievable OAR mean doses could be determined in advance by using the model solely to predict DVHs, without requiring the creation of an actual treatment plan. Although previous investigations showed the potential of RapidPlan to improve plan quality [5–7], the present study is the first to evaluate the accuracy of the dosimetric predictions. This is relevant because improved plan quality does not necessarily imply that accurate predictions were generated, whilst accurately modeling achievable OAR doses is an important prerequisite for using RapidPlan as a plan QA tool. The prediction accuracy was determined for mean OAR doses because such doses have been shown to correlate to late toxicity for HNC patients [22–26].

Since the created RapidPlan model was found able to accurately predict the achievable mean OAR doses (Fig. 6), it could be used to evaluate the quality of previously created (manually optimized) clinical plans. Under the test conditions, this suggested that 50 % of the evaluated plans contained a composite OAR for which the sparing could be improved. These predicted gains in plan quality were not unexpected since the RapidPlan model was made using an AIO plan library, and AIO plans have been previously shown to provide improved OAR sparing over manual interactively optimized plans, along with being optimized in a more consistent fashion (9). Consistent with this, only 4/20 AIO plans were rejected because oral cavity sparing could have been improved, while salivary gland and swallowing muscle sparing was acceptable in all plans. The predicted improvements in plan quality were validated by replanning the patient using the RapidPlan model. The agreement was consistent with the high R^2^ values between the predicted and achieved mean OAR doses, and thus demonstrated successful application of RapidPlan-predicted DVH metrics as a QA tool. It is important to note that the high-level correlation between predicted and achieved dosimetry also allows the RapidPlan model to be used to evaluate the plans included in the model library. Replanning sub-optimal plans would allow for the continuous and consistent improvement of the model library and the resulting plan quality.

Although on average, RapidPlan could accurately predict mean doses, predictions could deviate in the high-dose regions for OARs with both low (<20 Gy) and high (>40 Gy) mean doses (Fig. 7). For the latter, RapidPlan overestimated the amount of achievable OAR sparing, probably because RapidPlan placed the line objective horizontally in the region of OAR-PTV overlap. In plans with multiple dose levels, OARs that only overlap with the lower dose PTV get no optimization objectives to restrict doses above the prescribed dose for this PTV (Fig. 1). Although this may not be as important in organs where toxicity is correlated with mean OAR dose, it could be an issue when high dose volumes are considered predictors of toxicity. If in future releases of RapidPlan, line objectives are also modeled for the part of the OAR that overlaps with the PTV, correlations between predicted and achievable OAR DVHs should improve.

**Fig. 9:**
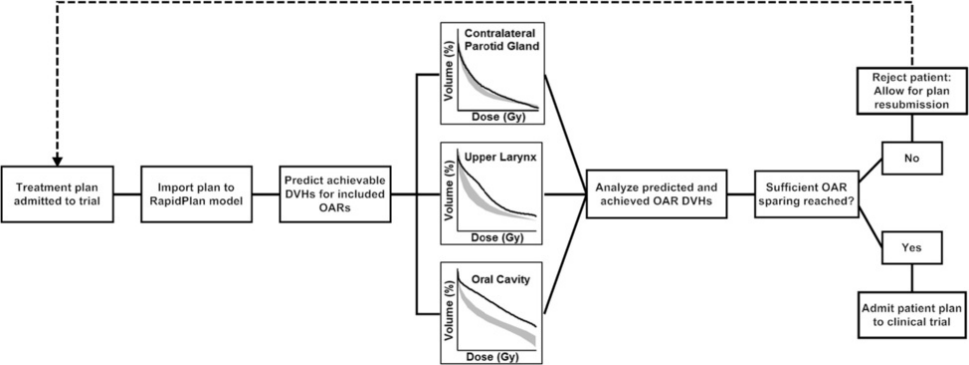
A flowchart proposing how the organ-at-risk (OAR) dose-volume histogram (DVH) predictions generated by a RapidPlan model could be used for fast plan quality assurance (QA) for clinical trials

Clinical trials often include treatment plans submitted by a large number of institutes. The difficulty of determining whether sufficient OAR sparing is reached for individual patients may lead to the inclusion of poor treatment plans, even though the magnitude of achieved OAR sparing may influence the outcome of the study [11, 27]. RapidPlan models that consist of a wide range of patient plans in which a consistently good level of OAR sparing was reached allow for fast, patient-specific evaluation of OAR DVHs for plans submitted to the study. Figure 9 suggests a workflow to quickly decide whether a submitted plan provides sufficient sparing based on its OAR-PTV geometry. In addition, the present results show the feasibility for clinical trials to supply participating centers with a RapidPlan model to ensure that all created plans are according to the specified guidelines. It is important to note that the validity of a RapidPlan model needs to be re-evaluated when dose prescriptions or optimization priorities change over time. Trial-specific model libraries may be required depending on the planning criteria.

Accurate DVH predictions made by RapidPlan models could similarly be used to benchmark the quality of plans that were created using alternative delivery techniques, such as proton therapy [28], without requiring the creation of photon plans. A comparable study was recently performed that evaluated the potential of an IMRT model from one institute and made using one IMRT technique to aid IMRT planning of another institute and predict achievable DVHs with another IMRT technique [18]. They found that a fixed gantry model could accurately predict the median dose of the parotid gland in Tomotherapy plans, which indicated that it was spared similarly by both institutions. In addition, predictions of median dose reductions to some OARs in the Tomotherapy plans could be achieved after replanning.

Potential limitations of the current study include the following. RapidPlan indicated that for some swallowing muscles, more training cases containing these structures were required to improve the model (Table 1). This may have influenced the predicted and obtained mean doses. All plans included in the model were created using the same number of arcs, and similar collimator angles and field sizes for most plans. If plans created using different field settings are evaluated, RapidPlan may predict OAR doses that are only realizable after changing the field settings. Additionally, different optimization and dose calculation algorithms were used to create the plans contained in the RapidPlan model library (PRO and AAA v10.0.28) and the RapidPlan plans (photon optimizer [PO] and AAA v13.5.33). Small improvements in OAR sparing when using newer algorithms were found previously [7]. Part of the gains achieved by RapidPlan may therefore be attributed to the use of the newer optimization and dose calculation algorithms, and in effect, the RapidPlan model predictions slightly underestimated the gains that could be achieved. Although our study showed good correlations between achieved and predicted OAR doses, determining the optimality of the plans included in the model library or of the resulting plans was beyond the scope of the present study. Future studies could incorporate retrospective RapidPlan QA of completed clinical trials. If a RapidPlan model were to be used as a planning QA tool for clinical trials, time and effort would need to be invested to ensure appropriate model composition and quality. Finally, if changes in treatment planning techniques lead to improvements in plan quality, these improved plans would need to be incorporated into updated RapidPlan libraries to ensure that the QA model remained state of the art, and of sufficiently high quality.

## Conclusions

The present study showed that RapidPlan models can accurately predict achievable mean doses for most OARs, enabling them to be used to benchmark the quality of existing plans. However, the predictions may be less accurate for OARs that overlap substantially with the PTVs and may not accurately reflect the shape of the DVH-line. Addressing these limitations should improve the prediction accuracy and the potential of RapidPlan for plan quality assessment.
